# Perfectionism and Identity Processes in Two Domains: Mediational Roles of Worry, Rumination, Indecisiveness, Shame, and Guilt

**DOI:** 10.3389/fpsyg.2019.01864

**Published:** 2019-08-23

**Authors:** Konrad Piotrowski

**Affiliations:** Psychology Institute, SWPS University of Social Sciences and Humanities, Poznań, Poland

**Keywords:** perfectionism, identity development, mediation, guilt, shame, indecisiveness, worry, rumination

## Abstract

The objective of the study was to assess the relationships between two dimensions of perfectionism, that is, perfectionistic strivings and perfectionistic concerns, and identity processes in the domains of future plans and education. It was hypothesized that such consequences of perfectionism as worry, rumination, indecisiveness, and guilt and shame proneness would be mediators of the perfectionism–identity relationship. A total of 696 students took part in the study (*M*_age_ = 26.74, *SD* = 7.56). It was found that perfectionistic strivings may promote the development of identity by supporting adaptive exploration and identity commitment. This relationship was only partly mediated. On the other hand, perfectionistic concerns were associated with significant difficulties with identity formation. This relationship was largely indirect, and indecisiveness proved to be the main mediator of the perfectionistic concerns–identity relationship. High indecisiveness, which disturbs decision-making processes, seems to explain why maladaptive perfectionists have problems with identity formation. These mediational effect was observed in both analyzed domains.

## Introduction

Personal identity is a personality structure that arises from identification with selected values, social roles, and lifestyles (Marcia, 1966). According to the neo-Eriksonian approach, characteristic for developmental psychology (Luyckx et al., 2011), optimal development of identity in adolescence and adulthood requires exploration processes, during which individuals can obtain information about themselves and about the environment, as well as make commitments in important domains, such as ideological, professional, and educational areas (Marcia, 1966). A stable sense of identity helps in making decisions, managing one’s life, and perceiving oneself as a unique individual (Luyckx et al., 2011). Hence, identity is an important determinant of quality of life (Karaś et al., 2015).

Luyckx et al. (2008c; for a review see Luyckx et al., 2011) described identity development as having two related cycles: (1) a *commitment formation cycle*, in which an individual explores broadly (a process called *exploration in breadth*: seeking new information on potential areas of commitment) and makes commitments (*commitment making* process: refers to enacting choices in different identity domains often as a result of exploration in breadth) and (2) a *commitment evaluation cycle*, in which the commitments undertaken are thoroughly assessed (this process is called *exploration in depth* and refers to gathering information about commitments in order to evaluate them), which opens the way to identification with these commitments (*identification with commitment*: the level of certainty with regard to commitments made). However, sometimes, mainly for individual reasons (e.g., high anxiety, depression), exploration processes take a maladaptive form called *ruminative exploration* (constant returning to ineffective ways of dealing with identity issues), and instead of supporting identity commitment, they may even hinder it (Luyckx et al., 2008b,c). Similarly, Meeus and Crocetti’s identity model (Crocetti et al., 2008) postulates that, after making a commitment, people perform two types of exploration: (1) *in-depth exploration* (similar to exploration in-depth from the Luyckx model), in which the individual focuses on better understanding of the area of commitment, and (2) *reconsideration of commitment*, based on comparing alternative decisions (if the alternatives are considered more satisfying, an individual may start to regret the decisions made and seek to change their identity). In these two models, identity formation in adolescence and adulthood is a continuous process of making commitments, assessing them, and changing (if they prove unsatisfactory) or identifying with them (if they meet an individual’s expectations). Research conducted within these two models showed that adaptive exploration processes focused on obtaining information (exploration in breadth, exploration in depth), and strong commitments and identification with them, are associated with better adaptation, while people with high ruminative exploration and strong reconsideration of commitment are characterized by lower quality of life, high anxiety, low self-esteem, and increased risk of mental problems (Luyckx et al., 2008c, d; Klimstra and Denissen, 2017; Piotrowski, 2018).

As a stable sense of identity is seen as an important predictor of adjustment and psychopathology (Klimstra and Denissen, 2017), numerous studies have focused on personality factors responsible for different identity formation trajectories in adolescence in adulthood. It was observed that such indicators of a low adjustment as high anxiety, neuroticism, depressive symptoms, and personality disorders are positively correlated with difficulties in identity formation (higher ruminative exploration and reconsideration of commitment, lower commitment and adaptive exploration processes; Crocetti et al., 2009; Klimstra et al., 2013; Claes et al., 2014; Bogaerts et al., 2018) while internal locus of control, sense of coherence, and good emotional regulation support coping with identity crisis (Luyckx et al., 2008a; Piotrowski and Brzeziñska, 2018). As identity formation requires making important decisions and evaluating them, it can be assumed that, in general, the personality factors that disrupt decision-making processes should at the same time hinder identity development (Vondracek et al., 1995). As one of the personality characteristics that strongly affects decision making is perfectionism (Brand and Altstötter-Gleich, 2008), it can be supposed that it should also influences the process of identity formation. Unfortunately, our knowledge of the links between perfectionism and identity formation is extremely limited. The present study aimed to obtain a better understanding of this relationship, to verify same previous observations, and to provide new knowledge of the role of perfectionism in identity formation processes in adulthood.

Perfectionism is a personality characteristic manifested in setting very high, often unrealistic standards and striving to realize them flawlessly. This tendency is often accompanied by subordinating self-evaluation to the achievement of these standards, fear of failure, and self-criticism (Frost et al., 1990; Hewitt and Flett, 1991; Hewitt et al., 2017). Most of the facets of perfectionism are believed to belong to two higher-order dimensions: *perfectionistic strivings* (setting high expectations, striving for perfection) and *perfectionistic concerns* (fear of failure, self-criticism; Stoeber and Otto, 2006). Although these two dimensions are positively correlated, when their specific impact is controlled for, they differ fundamentally. Perfectionistic strivings are positively associated with conscientiousness, positive affect, and active coping styles, while perfectionistic concerns are related to negative affect, depression, eating disorders, shame, anxiety, and personality pathology (Ashby et al., 2006; Stoeber and Otto, 2006; Stoeber et al., 2007; Dimaggio et al., 2015). As a result, perfectionistic strivings are considered as an adaptive facet of perfectionism supporting adjustment while perfectionistic concerns are related to increased psychopathology risk (Stoeber and Otto, 2006). Individual differences in the intensity of perfectionistic strivings and concerns are sometimes use as a base for distinguishing groups of adaptive (high strivings, low concerns) and maladaptive (high strivings, high concerns) perfectionists (Stoeber and Otto, 2006).

So far, only two studies have been conducted on the relationship between different aspects of perfectionism (i.e., strivings and concerns) and identity processes. Luyckx et al. (2008d) found that perfectionistic strivings were positively related to adaptive exploration (in breadth and depth), commitment making, and identification with commitment, and negatively to ruminative exploration. On the other hand, perfectionistic concerns were associated with lower commitment making and identification with commitment, and higher ruminative exploration. In turn, Piotrowski (2019) analyzed the relationship between mothers’ perfectionism and their sense of parental identity. His results support Luyckx’s observations. Perfectionistic strivings were positively associated with in-depth exploration (search for in-depth information about parenting, interest in the child), while perfectionistic concerns were positively associated with reconsideration of identity commitment in the parental domain (lack of parenting satisfaction, regret of decision on parenting). These results suggest that perfectionistic concerns may increase difficulties with identity formation, probably because people high on perfectionistic concerns, due to an excessively critical attitude toward themselves, are never satisfied with their current commitments. For this reason, their commitment is low, and a sense of confusion and insecurity is dominant.

These two studies on the perfectionism–identity relationship indicate that perfectionists, especially those high on perfectionistic concerns (the so-called maladaptive perfectionists; Stoeber and Otto, 2006), actually experience difficulties with identity development. However, none of these studies explained the potential mechanism of the influence of perfectionism on identity. As shown by many studies, perfectionism often influences adaptation and health in an indirect way.

A model that explains an impact of perfectionism on both mental and somatic health is the perfectionism cognition theory (Flett et al., 2016, 2018). According to this approach, perfectionism influences mental and somatic health through its close relationships with such cognitive characteristics as a high tendency to worry, rumination, and indecisiveness, resulting in higher stress and negative emotions (e.g., anxiety, shame, and guilt) experienced by perfectionists in everyday situations. This, in turn, directly leads to deterioration of functioning, increasing the risk of such problems as depression and sleeping difficulties (e.g., Ashby et al., 2006; Flaxman et al., 2017). Interestingly, similar factors were also observed to be associated with identity development. People who experience difficulties in the process of forming a stable sense of identity have a higher tendency to experience shame (Piotrowski, 2013), increased ruminations and anxiety (Luyckx et al., 2008c), and a tendency to worry (Gati et al., 2011). Depression, an affective disorder closely related to perfectionism (Hewitt et al., 1996) is also related to difficulties in the process of identity formation (Claes et al., 2014). It suggests that the perfectionism cognition theory may be useful in studying individual differences in identity development in adolescence and adulthood. This opens a space to integrate research on perfectionism and identity, which has not yet received an in-depth analysis. It is suggested here that cognitive and emotional consequences of perfectionism that make perfectionists extremely vulnerable to life feedback indicating that their lives are not going according to plan (Stoeber and Eismann, 2007; Flett et al., 2018) make it difficult for them to make definite identity commitments in different domains leading to prolonged sense of insecurity and identity diffusion.

### Research Problem and Hypotheses

This study aimed to provide further knowledge about the relationship between perfectionistic strivings and perfectionistic concerns, and the identity processes described in the models of Luyckx et al. (2008c) and Meeus and Crocetti (Crocetti et al., 2008). As recent studies show that different domains of identity development are weakly related and may develop differently (Piotrowski, 2018; Vosylis et al., 2018), the present study included separate analyses in the domains of future plans (Luyckx et al., 2008c) and of educational identity (Crocetti et al., 2008). This was to determine whether perfectionism and its cognitive and emotional consequences are related to identity processes in these domains in similar ways.

It was anticipated that perfectionistic strivings would be associated with higher adaptive exploration (exploration in breadth, and in depth) and with higher commitment and identification with commitment, while perfectionistic concerns would be associated primarily with difficulties in identity formation (positive associations with ruminative exploration and reconsideration of commitment, and negative associations with commitment processes).

The present study is the first one devoted to identification of mediating factors in the relationship of perfectionism and identity processes. It was anticipated that in line with the perfectionism cognition theory such consequences of perfectionism, especially of perfectionistic concerns, as worry, indecisiveness, and rumination, and proneness to experience such negative emotions as guilt and shame (Flett et al., 2016, 2018) would mediate the perfectionism–identity relationship. It was hypothesized that the direct relationship between perfectionism and identity (Luyckx et al., 2008d; Piotrowski, 2019) would significantly diminish when the above-mentioned cognitive and emotional consequences of perfectionism were taken into account.

## Materials and Methods

### Participants

The study included 696 participants, aged 18 to 42 years (*M* = 26.74, *SD* = 7.56). All participants were Polish, full-time or part-time psychology students, and Caucasian; 85% of the respondents were women. All participants were informed of the purpose of the study and about the voluntary nature of participation. The students received credits for participation, which they needed to complete their studies (the students at my University are also offered many other ways to receive credits in order to adhere to the principle of voluntary participation). All participants provided informed consent.

### Measures

#### Perfectionism

Three subscales from the Frost Multidimensional Perfectionism Scale were used (FMPS; Frost et al., 1990; Polish adaptation: Piotrowski and Bojanowska, 2019). Perfectionistic strivings were assessed using the Personal Standards subscale (seven items, e.g., *It is important to me that I be thoroughly competent in everything I do*), while perfectionistic concerns were assessed with the sum of two other subscales: Concern over Mistakes (nine items, e.g., *If I fail at work/school, I am a failure as a person*) and Doubts about Actions (four items, *Even when I do something very carefully, I often feel that it is not quite done right*). The remaining subscales were not included in the analyses as they do not constitute core facets of perfectionism (Stoeber and Otto, 2006). Answers were given on a five-point Likert scale, from *1-strongly disagree* to *5-strongly agree*. Cronbach’s alphas were 0.77 and 0.91, respectively.

#### Identity Processes

Identity processes in the future plans domain were assessed using the Dimensions of Identity Development Scale (DIDS; Luyckx et al., 2008c; Polish adaptation: Piotrowski and Brzezińska, 2017). The scale measures five processes: exploration in breadth (five items, e.g., *I try to find out which lifestyle would be good for me*), exploration in depth (five items, e.g., *I work out for myself if the aims I put forward in life really suit me*), ruminative exploration (five items, e.g., *I am doubtful about what I really want to achieve in life*), commitment making (five items, e.g., *I have decided on the direction I want to follow in my life*), and identification with commitment (five items, e.g., *My plans for the future match with my true interests and values*). Answers were given on a five-point Likert scale, from *1-strongly disagree* to *5-strongly agree*. Cronbach’s alphas were 0.79, 0.71, 0.87, 0.92, and 0.90, respectively. The second identity measure was the Utrecht-Management of Identity Commitments Scale, in the version used to measure educational identity (U-MICS; Crocetti et al., 2008; Polish adaptation: Karaś et al., 2013). The U-MICS measures three processes: commitment (five items, e.g., *My education makes me feel sure of myself*), in-depth exploration (five items, e.g., *I try to find out a lot about my education*), and reconsideration of commitment (three items, e.g., *I often think that a different education would make my life more interesting*). The answers were given on a five-point Likert scale, from *1-completely untrue* to *5-completely true*. Cronbach’s alphas were 0.89, 0.72, and 85, respectively.

#### Shame and Guilt

Proneness to shame and guilt was measured with the Personal Feelings Questionnaire-2 (PFQ-2; Harder and Zalma, 1990; Polish adaptation: Piotrowski and Brzezińska, 2017). The scale consists of a list of 22 adjectives describing emotional states, 10 of which assess proneness to feeling shame (e.g., *feeling humiliated, feeling laughable*), and 6 assess proneness to feeling guilt (e.g., *regret, intense guilt*); the other items were not included. The respondent indicates the frequency of experiencing individual emotional states on a scale from *0-never* to *4-continuously* or *almost continuously*. Cronbach’s alphas were 0.88 and 0.83, respectively.

#### Worry

The *Worry Domains Questionnaire-Short Form* (WDQ-SF; Stöber and Joormann, 2001; Piotrowski and Bojanowska, 2019) was used to measure the severity of the tendency to worry. The scale has been translated using the back-translation procedure. The tool consists of 10 items measuring the severity of worry in five areas: Relationships (e.g., *I worry that I will lose close friends*), Lack of Confidence, Aimless Future, Work, and Financial. All items were rated on a five-point scale, from *0-not at all* to *4-extremely*. As the DIDS questionnaire also concerns assessment of the future plans, two WDQ-SF items related to the future were not included in the analysis. The general score was used, being the sum of the eight remaining items. Cronbach’s alpha was 0.90.

#### Indecisiveness

Indecisiveness was measured with the Frost Indecisiveness Scale (FIS; Frost and Shows, 1993; Piotrowski and Bojanowska, 2019). The measure consists of 15 items measuring the intensity of general indecisiveness (e.g., *I try to put off making decisions*), assessed on a Likert scale from *1-strongly disagree* to *5-strongly agree*. Cronbach’s alpha was 0.91.

#### Rumination

The tendency to ruminate was measured by one of the scales of the shortened version of the Rumination-Reflection Questionnaire (RRQ; Trapnell and Campbell, 1999; Polish adaptation: Słowińska et al., 2014). The scale consists of six items measuring the tendency to anxiety evoked by involuntary concentration on one’s own experiences (e.g., *My attention is often focused on aspects of myself I wish I’d stop thinking about*). Items were rated on a Likert scale from *1-disagree*, to *5-strongly agree*. Cronbach’s alpha was 0.84.

### Statistical Analysis

Pearson’s correlation was used to evaluate bivariate correlations between all variables. The analyses were performed using SPSS 25. Then, a mediation analysis was carried out (Holmbeck, 1997) using path analysis. In the first step, the direct effect models (models A) were tested, in which the dimensions of perfectionism (strivings and concerns) were each direct predictors of identity processes from five dimensions from the Luyckx model (the future plans domain) or three dimensions from the Meeus model (the educational domain). In the second step, the full mediation models (models B) that did not assume direct relationships between perfectionism and identity were tested. Mediators were guilt, shame, worry, indecisiveness, and rumination. Finally, in the third step a partial-mediation models (models C) were tested, in which the significant direct associations between perfectionism and identity were also included in the full-mediation models.

Mediation analysis was carried out using the Mplus 7.3 software (Muthén and Muthén, (1998-2012). Because the multivariate distribution deviated somewhat normality, the Satorra-Bentler scaled χ^2^ (SBS-χ^2^; Satorra and Bentler, 1994), whose lower value indicates a better model fit, was used. Assessment of fit used the following indices (Hu and Bentler, 1999): the Comparative Fit Index (CFI), whose value should be higher than 0.95, the Root Mean Square Error of Approximation (RMSEA), whose value should be lower than 0.06, and the Standardized Root Mean Square Residual (SRMR), whose value should be less than 0.08. To assess changes in the fit of subsequent models, the significance of changes in the fit parameters was checked. Based on Chen (2007), it was assumed that the parameters changed significantly when ΔCFI ≥ 0.010, ΔRMSEA ≥ 0.015, and ΔSRMR ≥ 0.010.

Because shame and guilt are similar emotions, to assess their specificity, it is recommended to carry out analyses after excluding the common variance of these variables (so-called *shame-free guilt* and *guilt-free shame*; Tangney et al., 1996) and to use regression residuals. This procedure was applied in all analyses.

## Results

### Preliminary Analysis

Table 1 shows the correlations between perfectionism, the mediators, and identity. In general, the results were in line with the predictions and findings of other studies (Luyckx et al., 2008d; Piotrowski, 2019). Perfectionistic strivings correlated positively with adaptive exploration variables (DIDS exploration in breadth and in depth, U-MICS in-depth exploration) and positively with the dimensions of identity commitment. Perfectionistic concerns correlated positively with difficulties in identity development (positive correlations with ruminative exploration and reconsideration of commitment). The dimensions of perfectionism were positively correlated with the mediators, but for perfectionistic strivings, the relationships were much weaker than for perfectionistic concerns. Guilt, shame, worry, indecisiveness, and rumination were generally associated with greater difficulties in identity development, but there were significant differences in the strength of the relationships between the variables. The strongest connections with identity processes were with worry, indecisiveness, and rumination.

**TABLE 1:** Correlations between the analyzed variables.

	**1**	**2**	**3**	**4**	**5**	**6**	**7**	**8**	**9**	**10**	**11**	**12**	**13**	**14**	**15**
(1) PS	–	0.32^∗∗∗^	0.07	–0.05	0.10^∗∗^	–0.04	0.08^∗^	0.13^∗∗^	0.22^∗∗∗^	–0.01	0.19^∗∗∗^	0.21^∗∗∗^	0.15^∗∗∗^	0.17^∗∗∗^	0.01
(2) PC		–	0.11^∗∗^	0.34^∗∗∗^	0.62^∗∗∗^	0.57^∗∗∗^	0.57^∗∗∗^	0.13^∗∗^	0.13^∗∗^	0.44^∗∗∗^	–0.21^∗∗∗^	–0.24^∗∗∗^	–0.19^∗∗∗^	0.02	0.31^∗∗∗^
(3) Guilt			–	–0.75^∗∗∗^	0.11^∗∗^	0.13^∗∗^	0.16^∗∗∗^	0.05	0.01	0.09^∗^	–0.01	–0.02	−0.08^∗^	0.04	0.06
(4) Shame				−	0.35^∗∗∗^	0.27^∗∗∗^	0.21^∗∗∗^	–0.02	0.03	0.25^∗∗∗^	–0.19^∗∗∗^	–0.19^∗∗∗^	−0.09^∗^	–0.05	0.15^∗∗∗^
(5) Worry					–	0.58^∗∗∗^	0.53^∗∗∗^	0.11^∗∗^	0.17^∗∗∗^	0.49^∗∗∗^	–0.24^∗∗∗^	–0.23^∗∗∗^	–0.19^∗∗∗^	0.05	0.30^∗∗∗^
(6) Indecisiveness						–	0.53^∗∗∗^	0.04	0.02	0.54^∗∗∗^	–0.42^∗∗∗^	–0.41^∗∗∗^	–0.31^∗∗∗^	–0.03	0.30^∗∗∗^
(7) Rumination							−	0.18^∗∗∗^	0.21^∗∗∗^	0.43^∗∗∗^	–0.22^∗∗∗^	–0.22^∗∗∗^	–0.22^∗∗∗^	0.06	0.16^∗∗∗^
(8) EB								–	0.44^∗∗∗^	0.18^∗∗∗^	0.18^∗∗∗^	0.15^∗∗∗^	0.09^∗^	0.27^∗∗∗^	–0.01
(9) ED									–	0.09^∗^	0.23^∗∗∗^	0.38^∗∗∗^	0.19^∗∗∗^	0.41^∗∗∗^	–0.01
(10) RE										–	–0.62^∗∗∗^	–0.55^∗∗∗^	–0.40^∗∗∗^	–0.04	0.50^∗∗∗^
(11) CM											–	0.72^∗∗∗^	0.42^∗∗∗^	0.22^∗∗∗^	–0.37^∗∗∗^
(12) IC												–	0.51^∗∗∗^	0.30^∗∗∗^	–0.43^∗∗∗^
(13) COM/edu													–	0.46^∗∗∗^	–0.44^∗∗∗^
(14) EXP/edu														–	–0.19^∗∗∗^
(15) REC/edu															–

*^∗^*p* < 0.05, ^∗∗^*p* < 0.01, ^∗∗∗^*p* < 0.001. PS, perfectionistic strivings; PC, perfectionistic concerns; EB, exploration in breadth; ED, exploration in depth; RE, ruminative exploration; CM, commitment making; IC, identification with commitment; COM/edu, commitment in the educational domain; EXP/edu, in-depth exploration in the educational domain; REC/edu, reconsideration of commitment in the educational domain.*

As previous studies have shown that some of the variables analyzed here are related to age and gender (e.g., Luyckx et al., 2008a), additional analyses were carried out to verify this. Correlation analysis showed that participants’ age correlated significantly negatively with perfectionistic concerns (−0.21), shame (−0.20), worry (−0.27), indecisiveness (−0.28), rumination (−0.31), ruminative exploration (−0.25), and reconsideration of commitment (−0.19), and positively with U-MICS commitment (0.19), DIDS commitment making (0.23), and DIDS identification of commitment (0.18). To assess the relationship between the analyzed variables and sex, a multivariate analysis of variance with sex as a factor was performed. The results revealed that women had slightly higher scores on exploration in depth (DIDS), U-MICS commitment, and the U-MICS in-depth exploration scales. Based on these results, age and gender were controlled in all mediation models.

### Perfectionism and Identity Processes in the Future Plans Domain

The first step was the direct effects model test (A), in which the two dimensions of perfectionism were treated as predictors of the five identity processes from the Luyckx model (Luyckx et al., 2008c). This model was saturated and thus perfectly fitted to the data. However, because perfectionistic concerns were not significantly related to exploration in breadth and in depth, the paths coefficients in those cases were set to 0, which allowed calculation of the fit indices. The model created in this way was very well fitted to the data: SBS-χ^2^ (*df* = 2, *N* = 696) = 4.38, *p* > 0.05, CFI = 0.99, RMSEA = 0.04, SRMR = 0.02. Perfectionistic strivings were positively associated with exploration in breadth (β = 0.13, *p* < 0.01), exploration in depth (β = 0.21, *p* < 0.001), commitment making (β = 0.28, *p* < 0.001), and identification with commitment (β = 0.32, *p* < 0.001), and negatively with ruminative exploration (β = −0.16, *p* < 0.001). Perfectionistic concerns were related negatively with commitment making (β = −0.27, *p* < 0.001) and with identification with commitment (β = −0.33, *p* < 0.001), and positively with ruminative exploration (β = 0.45, *p* < 0.001). The model explained 2% of the variance in exploration in breadth, 6% of the variance in exploration in depth, 24% of the variance in ruminative exploration, 15% of the variance in commitment making, and 18% of the variance in identification with commitment.

In the next step, the full-mediation model (B) was tested, where guilt, shame, worry, indecisiveness, and rumination were placed as mediators. Both dimensions of perfectionism were treated as predictors of the mediators, and the mediators as direct predictors of identity dimensions. Model B was also well fitted to the data, SBS-χ^2^ (*df* = 10, *N* = 696) = 47.15, *p* < 0.001, CFI = 0.99, RMSEA = 0.07, SRMR = 0.03. Finally, in the third step, model C was evaluated (a partial mediation model in which the direct relations between perfectionism and identity were added; paths that were insignificant in model A were set to 0). The partial mediation model proved to be better fitted than model B, SBS-χ^2^ (*df* = 2, *N* = 696) = 2.49, *p* > 0.05, CFI = 1.00, RMSEA = 0.02, SRMR < 0.01 (ΔCFI = 0.01, ΔRMSEA = 0.05, ΔSRMR = 0.03). Model C explained 6% of the variance in exploration in breadth, 11% of the variance in exploration in depth, 37% of the variance in ruminative exploration, 23% of the variance in commitment making, and 23% of the variance in identification with commitment. Taking all this into account, it was assumed that the partial mediation model (C) was the most optimal. The direct relations between variables from model C are shown in Table 2.

**TABLE 2:** Standardized regression coefficients in the partial-mediation model (C) with the DIDS dimensions as dependent variables (the domain of the future plan).

**Direct relationships between perfectionism dimensions (independent variables) and mediators**					
	**Guilt β**	**Shame β**	**Worry β**	**Indecisiveness β**	**Rumination β**
Perfectionistic strivings	0.04	–0.17^∗∗∗^	–0.10^∗∗^	–0.24^∗∗∗^	–0.07
Perfectionistic concerns	0.11^∗∗^	0.36^∗∗∗^	0.62^∗∗∗^	0.61^∗∗∗^	0.45^∗∗∗^
	*R*^2^ = 0.02	*R*^2^ = 0.16	*R*^2^ = 0.42	*R*^2^ = 0.40	*R*^2^ = 0.28

**Direct relationships between the mediators and the identity processes (dependent variables)**					
	**Exploration in breadth β**	**Exploration in depth β**	**Ruminative exploration β**	**Commitment making β**	**Identification with commitment β**

Shame	−0.17^∗^	−0.17^∗^	0.18^∗^	–0.14	−0.17^∗^
Guilt	–0.12	−0.17^∗^	0.15^∗^	–0.09	–0.13
Worry	0.10	0.19^∗∗∗^	0.15^∗∗^	0.04	0.07
Indecisiveness	–0.06	−0.13^∗^	0.27^∗∗∗^	–0.35^∗∗∗^	–0.31^∗∗∗^
Rumination	0.21^∗∗∗^	0.21^∗∗∗^	0.10^∗∗^	0.02	0.01

**Direct relationships between the perfectionism dimensions (independent variables) and the identity processes (dependent variables) when the mediators were included in the model**					
Perfectionistic strivings	0.10^∗^	0.18^∗∗∗^	–0.05	0.18^∗∗∗^	0.22^∗∗∗^
Perfectionistic concerns	0.00	0.00	0.06	–0.01	−0.10^∗^
Total variance of the identity processes explained by model C	*R*^2^ = 0.06	*R*^2^ = 0.11	*R*^2^ = 0.37	*R*^2^ = 0.23	*R*^2^ = 0.23

*^∗^*p* < 0.05, ^∗∗^*p* < 0.01, ^∗∗∗^*p* < 0.001. Paths from the control variables (age, gender) were omitted for reasons of clarity. DIDS, Dimensions of Identity Development Scale.*

Perfectionistic strivings were significantly negatively related to shame, worry, and indecisiveness. In turn, perfectionistic concerns were positively associated with all mediators, with the strongest relationships observed for worry and indecisiveness. The results also revealed the suppressor effect described in the literature on perfectionism (Stoeber and Damian, 2016). If the common variance of perfectionistic strivings and perfectionistic concerns is not controlled, as in the bivariate correlations (Table 1), the positive impact of perfectionistic strivings on functioning does not reveal itself or is weakened, because strivings are positively related to perfectionistic concerns, whose negative impact is usually stronger. Only controlling for the common variance, as in the path analysis, allows the adaptive function of perfectionistic strivings to be revealed, as can be seen in Table 2. The observed relationships of the mediators and the dimensions of identity showed an interesting diversity. Shame, guilt, and indecisiveness were negatively associated with the dimensions of adaptive exploration (in breadth, in depth), while worry and rumination were positively associated with them. At the same time, all mediators were significantly positively associated with ruminative exploration. Among the mediators, indecisiveness, and shame to a lesser degree, were also associated with lower commitment making and identification with commitment. It is worth noting that indecisiveness was more strongly associated than the other mediators with both ruminative exploration (positively) and with two dimensions of commitment (negatively).

After taking into account the mediators, the initial, negative relationship between perfectionistic strivings and ruminative exploration was completely reduced. Relationships with other dimensions of identity remained significant, but in two cases they were clearly weakened: exploration in breadth, from 0.32 to 0.10, and identification with commitment, from 0.36 to 0.22. In the case of perfectionistic concerns, their initial relationship with ruminative exploration and commitment making was completely reduced, while the negative relationship with identification with commitment remained significant, although it was weakened (from −0.33 to −0.10). Table 3 shows the indirect effects of perfectionism on the DIDS identity dimensions via the mediators. The main observation is the role of indecisiveness, which was the most important mediator among those analyzed.

**TABLE 3:** Indirect effects of the perfectionism dimensions on the DIDS identity processes via the analyzed mediators.

	**EB**	**ED**	**ER**	**CM**	**IC**
	**Indirect effect**	**Indirect effect**	**Indirect effect**	**Indirect effect**	**Indirect effect**
PS (total indirect)	–	–	–0.11^∗∗∗^	0.10^∗∗∗^	0.09^∗∗∗^
PS→guilt	–	–	–	–	–
PS→shame	–	0.03^∗^	−0.03^∗^	–	–
PS→worry	–	−0.02^∗^	−0.01^∗^	–	–
PS→indecisiveness	–	0.03^∗^	–0.07^∗∗∗^	0.08^∗∗∗^	0.07^∗∗∗^
PS→rumination	–	–	–	–	–
PC (total indirect)	–	–	0.39^∗∗∗^	–0.25^∗∗∗^	–0.22^∗∗∗^
PC→guilt	–	–	–	–	–
PC→shame	–	–	0.06^∗^	–	−0.06^∗^
PC→worry	–	–	0.09^∗∗^	–	–
PC→indecisiveness	–	–	0.17^∗∗∗^	–0.22^∗∗∗^	–0.19^∗∗∗^
PC→rumination	–	–	0.05^∗^	–	–

*For reasons of clarity only the significant effects are presented. DIDS, Dimensions of Identity Development Scale; PS, perfectionistic strivings; PC, perfectionistic concerns; EB, exploration in breadth; ED, exploration in depth; RE, ruminative exploration; CM, commitment making; IC, identification with commitment; COM/edu, commitment in the educational domain; EXP/edu, in-depth exploration in the educational domain; REC/edu, reconsideration of commitment in the educational domain.*

### Perfectionism and Identity Formation in the Educational Domain

Model A, as in the previous case, was saturated, but because perfectionistic concerns were not significantly associated with in-depth exploration, the value of this parameter was set to 0. The model thus created proved to be very well fitted to the data: SBS-χ^2^ (*df* = 1, *N* = 696) = 0.42, *p* > 0.05, CFI = 1.00, RMSEA < 0.01, SRMR < 0.01. Perfectionistic strivings were positively associated with commitment (β = 0.22, *p* < 0.001) and in-depth exploration (β = 0.17, *p* < 0.001), and negatively with reconsideration of commitment (β = −0.08, *p* < 0.05). Perfectionistic concerns were negatively connected with commitment (β = −0.22, *p* < 0.001) and positively with reconsideration of commitment (β = 0.31, *p* < 0.001). The dimensions of perfectionism explained 11% of the variance in commitment, 4% of the variance in in-depth exploration, and 12% of the variance in reconsideration of commitment.

In the next step, a full-mediation model (B) was tested, which was also very well suited to the data, SBS-χ^2^ (*df* = 6, *N* = 696) = 28.33, *p* < 0.001, CFI = 0.99, RMSEA = 0.07, SRMR = 0.02. Finally, in the third step, the partial mediation model (C) was evaluated, in which a direct relationship between perfectionism and identity was added (the path that was insignificant in model A was set to 0). The partial mediation model proved to be better fitted to the data than model B, SBS-χ^2^ (*df* = 1, *N* = 696) = 0.74, *p* > 0.05, CFI = 1.00, RMSEA = 0.02, SRMR < 0.01 (ΔCFI = 0.01, ΔRMSEA = 0.05, ΔSRMR = 0.02). Model C explained 15% of the variance in commitment, 5% of the variance in in-depth exploration, and 16% of the variance in reconsideration of commitment. The path coefficients in model C are shown in Table 4. For perfectionistic strivings, the strength of the direct relationships with identity dimensions was similar to that of model A, which indicates that for perfectionistic strivings, strong mediation did not occur. For perfectionistic concerns, their initial negative relationship with commitment was completely reduced, and for reconsideration of commitment it remained significant, although weakened (from 0.31 to 0.13).

**TABLE 4:** Standardized regression coefficients in the partial-mediation model (C) with the U-MICS dimensions as dependent variables (the educational domain).

**Relationships between perfectionism dimensions (independent variables) and mediators**					
	**Guilt β**	**Shame β**	**Worry β**	**Indecisiveness β**	**Rumination β**
Perfectionistic strivings	0.04	0.17^∗∗∗^	0.10^∗∗^	−0.24^∗^	–0.07
Perfectionistic concerns	0.11^∗∗^	0.36^∗∗∗^	0.62^∗∗∗^	0.61^∗∗∗^	0.45^∗∗∗^
	*R*^2^ = 0.02	*R*^2^ = 0.16	*R*^2^ = 0.42	*R*^2^ = 0.40	*R*^2^ = 0.28

**Relationships between mediators and identity processes (dependent variables)**					

	**Commitment β**		**In-depth exploration β**	**Reconsideration of commitment β**	

Guilt	–0.12		–0.12	0.14	
Shame	–0.09		−0.16^∗^	0.13	
Worry	0.04		0.11^∗^	0.11^∗^	
Indecisiveness	–0.19^∗∗^		–0.05	0.14^∗^	
Rumination	–0.07		0.08	−0.10^∗^	

**Direct relationships between perfectionism dimensions (independent variables) and identity processes when the mediators were included (partial-mediation)**					

Perfectionistic strivings	0.17^∗∗∗^		0.15^∗∗∗^	–0.03	
Perfectionistic concerns	–0.06		0.01	0.13^∗^	
Total variance of the identity processes explained by the model C	*R*^2^ = 0.15		*R*^2^ = 0.05	*R*^2^ = 0.16	

*^∗^*p* < 0.05, ^∗∗^*p* < 0.01, ^∗∗∗^*p* < 0.001. Regression paths from the control variables (age, gender) were omitted for reasons of clarity. U-MICS, Utrecht-Management of Identity Commitments Scale.*

Furthermore, in the educational domain, some of the mediators were positively associated with in-depth exploration (worry), while others were negatively related (shame). Among the mediators, indecisiveness was the only significant specific predictor of lower commitment and, together with worry, was a predictor of higher reconsideration of commitment. On the other hand, rumination was negatively related to reconsideration of commitment.

Table 5 presents indirect effects observed in model C. The indirect effect of perfectionistic strivings on commitment and reconsideration of commitment was exerted mainly through the level of indecisiveness. Indecisiveness also explained the negative relationship of perfectionistic concerns and commitment.

**TABLE 5:** Indirect effects of the perfectionism dimensions on the U-MICS identity processes via the analyzed mediators.

	**Commitment**	**In-depth exploration**	**Reconsideration of commitment**
	**Indirect effect**	**Indirect effect**	**Indirect effect**
PS (total indirect)	0.06^∗∗^	–	–0.06^∗∗^
PS→guilt	–	–	–
PS→shame	–	–	–
PS→worry	–	–	–
PS→indecisiveness	0.05^∗∗^	–	−0.03^∗^
PS→rumination	–	–	–
PC (total indirect)	–0.17^∗∗∗^	–	0.17^∗∗∗^
PC→guilt	–	–	
PC→shame	–	−0.06^∗^	
PC→worry	–	0.07^∗^	0.07^∗^
PC→indecisiveness	–0.12^∗∗∗^	–	0.09^∗^
PC→rumination	–	–	−0.05^∗^

*For reasons of clarity only the significant effects are presented. U-MICS, Utrecht-Management of Identity Commitments Scale.*

## Discussion

The present study focused on the relationships between perfectionism and identity. It was suggested that perfectionistic strivings would support coping with identity issues (higher commitment and adaptive exploration, lower ruminative exploration and reconsideration of commitment) while perfectionistic concerns would be a risk factor in this processes increasing the risk for identity diffusion (low commitment and adaptive exploration, higher ruminative exploration and reconsideration of commitment). The findings confirm these hypotheses and previous observations (Luyckx et al., 2008d; Piotrowski, 2019). Perfectionistic strivings seem to support the formation of a stable sense of identity by stimulating adaptive exploration and making identity commitments. This may be motivated by the desire to realize an ideal vision of oneself. Perfectionistic concerns, on the contrary, constitute an important risk factor in the development of identity, making it difficult to create a stable sense of identity and leading to uncertainty and confusion. Depending on the level of both dimensions in the perfectionist (i.e., adaptive and maladaptive perfectionists; Stoeber and Otto, 2006), we can expect different effects on identity development.

It was also suggested that cognitive processes (rumination, worry, and indecisiveness) and negative emotional experiences characteristic for perfectionists (shame and guilt) can be responsible for the observed relationships between perfectionism and identity processes (Luyckx et al., 2008d; Piotrowski, 2019). This assumption was supported to some extend but important differences between perfectionistic strivings and perfectionistic concerns were observed.

The results of the mediation analysis suggest that the relationship between perfectionistic strivings and identity is mainly direct, and the very fact that some people set very ambitious and demanding goals facilitates coping with an identity crisis. Admittedly, it has been observed that perfectionistic strivings also contribute to better cognitive and emotional functioning (i.e., lower shame proneness, worry, and indecisiveness), which indirectly support identity formation, but this indirect effect does not seem to play a key role here. Similarly, Chang (2006) observed that stress mediates the relationship between perfectionistic concerns (but not strivings) and well-being and Gnilka et al. (2012) showed that coping strategies mediate between perfectionistic concerns (but not strivings) and anxiety. Those results support treating perfectionistic strivings as a specific aspect of perfectionism that can have a positive and mainly direct impact on functioning including coping with identity issues.

The mediators analyzed, however, explained to a large extent the negative impact of perfectionistic concerns on identity. This observation is in line with the perfectionism cognition theory and supports using this approach for studying the link between perfectionism and identity development in adulthood. Perfectionistic concerns are the dark side of perfectionism and are not prominent in every perfectionist (Stoeber and Otto, 2006). Setting very high expectations (perfectionistic strivings) is, however, often associated with a strong fear that these standards will not be achieved, which will lead to criticism, failure, ridicule, and rejection (so called *maladaptive perfectionism*). Perfectionistic concerns lead to characteristic changes in the areas of cognitive and emotional functioning (Flett et al., 2016). Perfectionists with high concerns display a characteristic tendency to long-term worry about the future consequences of their actions, difficulties in making decisions, persisting states of self-reflection and ruminations, and often experience negative emotions such as here analyzed shame and guilt. These tendencies intensify and prolong stress in everyday situations and are seen as basic to the psychopathology of perfectionists (Flett et al., 2016). The obtained results show that these various consequences of perfectionistic concerns may have different, often contradictory, effects on the dynamics of identity processes, meaning that perfectionists can get trapped in contradictory tendencies and motivations, unable to make stable identity commitments. On the one hand, perfectionistic concerns seem to limit the processes of adaptive in-breadth and in-depth exploration. This results from perfectionists’ indecisiveness and their tendencies to experience guilt and shame. The desire to avoid these negative emotions and confrontation with one’s own inability to make decisions may lead to the reluctance of perfectionists to look for alternative ways of development and asking themselves questions about their own identity and in-depth assessment of their commitments. On the other hand, the tendency of perfectionists to be afraid of problems in the future (worry) and ruminating, that is, remembering mistakes from the past, push them in the opposite direction, to just engaging in processes of in-breadth and in-depth exploration, perhaps to make sure that the path taken is appropriate to avoiding anticipated problems in the future. These contradictory motives can lead perfectionists to a state of inability to make any identity decisions and returning to the same ineffective ways of dealing with identity issues. At the same time, the same inclinations (primarily indecisiveness) make it difficult to escape this vicious cycle of exploration, making it difficult to undertake and identify with commitments. This results in high ruminative exploration, uncertainty as to who one is (low commitment), and low identification with the undertaken decisions (lower identification with commitment, higher reconsideration of commitment).

This study was also the first to analyze the relationship between perfectionism and identity processes in the educational domain. The results suggest that these relationships are similar to those of other identity domains such as the future plans and parental domains studied earlier (Luyckx et al., 2008d; Piotrowski, 2019). This finding suggesting that the perfectionism-identity relationship works similar in different domains supports the global rather than the domain-specific approach to identity assessment (Vosylis et al., 2018). However, one should bear in mind that the educational domain, like the future plans and parental domains, studied by Luyckx et al. (2011) and Piotrowski (2019), belongs to the broader category of ideological domains. It cannot be ruled out that if relational domains were included, the results could be different. It is suggested to explore this issue in future research.

The relationships between perfectionism, cognitive and emotional functioning, and identity formation described in the present article also have clinical value, especially for clinical psychology and psychotherapy. Perfectionism is not a disorder but it is a transdiagnostic process involved in occurrence and maintenance of many mental health problems such as anorexia, bulimia, depression, bipolar disorder, anxiety disorders, and personality disorders (Egan et al., 2011). As recent studies show, difficulties with formation of a clear and stable sense of identity during adolescence and early adulthood is also involved in psychopathology occurrence. Recently, van Doeselaar et al. (2018) showed that not only is identity related to depression but also that stronger identity commitments predict a decrease in depressive symptoms in adolescents. The results presented here and the results obtained earlier by Luyckx et al. (2008d) might suggest then that identity development processes may mediate between trait perfectionism and psychopathology occurrence. This issue should be studied in the future as it could help us better understand the perfectionism-psychopathology link. Another implication of the study is related to psychotherapy of perfectionism and mental health problems related to perfectionism. The difficulties experienced by perfectionists have many sources. The presented results suggest two important things related to this issue. Firstly, the important source of difficulties in maladaptive perfectionists is their inability to making decisions and stress resulting from this decisional stuck. Secondly, even the influence of maladaptive perfectionism on functioning is not uniform. Even the well-known cognitive and emotional results of perfectionism, such as worry and shame proneness, can have a different impact on particular developmental processes and in a case of the particular patient it is important to diagnose and properly address this issues during the intervention program.

### Limitations

Although the present study provided new knowledge, its limitations must also be considered. First, the study mainly included women. Although there were few differences between them and men, and gender was controlled in structural equation models, caution should be exercised in generalizing the results. Second, the study was cross-sectional and requires verification in longitudinal studies. The results of this cross-sectional study do not exclude that other models can also be true. Hence, the observed relationships, even if theoretically justified, should not be treated as a definitive proof that the relations are exactly as shown – they should only be treated as plausible and verified in future longitudinal studies. Third, the mediators chosen were quite homogeneous and it is worth considering more diverse variables in subsequent studies. This could be why mediation effects were less visible for perfectionistic strivings. It is worth conducting further analyses with another set of mediators.

## Conclusion

Perfectionistic strivings and perfectionistic concerns seem to have different effects on identity development. The obtained results indicate that the positive impact of perfectionistic strivings is largely direct, while the negative impact of perfectionistic concerns seems to be predominantly mediated by cognitive and emotional difficulties resulting from perfectionism. High indecisiveness seems to be the important factor responsible for problems with identity formation among perfectionists, which disturbs decision-making processes.

## Data Availability

The datasets generated and/or analyzed in this study are available from the corresponding author.

## Ethics Statement

The studies involving human participants were reviewed and approved by the Departmental Ethics Committee, University of Social Sciences and Humanities, Poznañ, Poland. The patients and participants provided their written informed consent to participate in this study.

## Author Contributions

The author confirms being the sole contributor of this work and has approved it for publication.

## Conflict of Interest Statement

The author declares that the research was conducted in the absence of any commercial or financial relationships that could be construed as a potential conflict of interest.

## References

[B1] Ashby J. S. Rice K. G. Martin J. L. (2006). Perfectionism, shame, and depressive symptoms. *J. Couns. Dev.* 84 148–156. 10.1002/j.1556-6678.2006.tb00390.x

[B2] Bogaerts A. Claes L. Verschueren M. Bastiaens T. Kaufman E. A. Smits D. (2018). The dutch self-concept and identity measure (SCIM): factor structure and associations with identity dimensions and psychopathology. *Personal. Individ. Differ.* 123 56–64. 10.1016/j.paid.2017.11.007

[B3] Brand M. Altstötter-Gleich C. (2008). Personality and decision-making in laboratory gambling tasks – evidence for a relationship between deciding advantageously under risk conditions and perfectionism. *Personal. Individ. Differ.* 45 226–231. 10.1016/j.paid.2008.04.003

[B4] Chang E. C. (2006). Perfectionism and dimensions of psychological well-being in a college student sample: a test of a stress-mediation model. *J. Soc. Clin. Psychol.* 25 1001–1022. 10.1521/jscp.2006.25.9.1001

[B5] Chen F. F. (2007). Sensitivity of goodness of fit indexes to lack of measurement invariance. *Struct. Equ. Modeling* 14 464–504. 10.1080/10705510701301834

[B6] Claes L. Luyckx K. Bijttebier P. (2014). Non-suicidal self-injury in adolescents: prevalence and associations with identity formation above and beyond depression. *Personal. Individ. Differ.* 6 101–104. 10.1016/j.paid.2013.12.019

[B7] Crocetti E. Klimstra T. Keijsers L. Hale W. W. Meeus W. (2009). Anxiety trajectories and identity development in adolescence: a five-wave longitudinal study. *J. Youth Adolesc.* 38 839–849. 10.1007/s10964-008-9302-y 19636785

[B8] Crocetti E. Rubini M. Meeus W. (2008). Capturing the dynamics of identity formation in various ethnic groups: development and validation of a three-dimensional model. *J. Adolesc.* 31 207–222. 10.1016/j.adolescence.2007.09.002 17959238

[B9] Dimaggio G. Lysaker P. H. Calarco T. Pedone R. Marsigli N. Riccardi I. (2015). Perfectionism and personality disorders as predictors of symptoms and interpersonal problems. *Am. J. Psychother.* 69 317–330. 10.1176/appi.psychotherapy.2015.69.3.317 26414312

[B10] Egan S. J. Wade T. D. Shafran R. (2011). Perfectionism as a transdiagnostic process: a clinical review. *Clin. Psychol. Rev.* 31 203–212. 10.1016/j.cpr.2010.04.009 20488598

[B11] Flaxman P. E. Stride C. B. Söderberg M. Lloyd J. Guenole N. Bond F. W. (2017). Relationships between two dimensions of employee perfectionism, postwork cognitive processing, and work day functioning. *Eur. J. Work Organ. Psychol.* 27 56–69. 10.1080/1359432X.2017.1391792

[B12] Flett G. L. Hewitt P. L. Nepon T. Besser A. (2018). “Perfectionism cognition theory: the cognitive side of perfectionism,” in *The Psychology of Perfectionism. Theory, Research, Applications*, ed. Stoeber J. (London: Routledge), 89–110. 10.4324/9781315536255-7

[B13] Flett G. L. Nepon T. Hewitt P. L. (2016). “Perfectionism, worry, and rumination in health and mental health: a review and a conceptual framework for a cognitive theory of perfectionism,” in *Perfectionism, Health, and Well-Being*, eds Sirois F. M. Molnar D. S. (New York, US: Springer).

[B14] Frost R. O. Marten P. Lahart C. Rosenblate R. (1990). The dimensions of perfectionism. *Cognit. Ther. Res.* 14 449–468.

[B15] Frost R. O. Shows R. L. (1993). The nature and measurement of compulsive indecisiveness. *Behav. Res. Ther.* 31 683–692. 8216169 10.1016/0005-7967(93)90121-a

[B16] Gati I. Gadassi R. Saka N. Hadadi Y. Ansenberg N. Friedmann R. (2011). Emotional and personality-related aspects of career decision-making difficulties: facets of career indecisiveness. *J. Career Assess.* 19 3–20. 10.1177/1069072710382525

[B17] Gnilka P. B. Ashby J. S. Noble C. M. (2012). Multidimensional perfectionism and anxiety: differences among individuals with perfectionism and tests of a coping-mediation model. *J. Couns. Dev.* 90 427–436. 10.1002/j.1556-6676.2012.00054.x

[B18] Harder D. H. Zalma A. (1990). Two promising shame and guilt scales: a construct validity comparison. *J. Personal. Assess.* 55 729–745. 10.1080/00223891.1990.9674108 2280336

[B19] Hewitt P. L. Fleet G. L. Mikail S. F. (2017). *Perfectionism. A Relational Approach to Conceptualization, Assessment, and Treatment.* New York, NY: Guilford Press, 10.1080/00223891.1990.9674108

[B20] Hewitt P. L. Flett G. L. (1991). Perfectionism in the self and social contexts: conceptualization, assessment, and association with psychopathology. *J. Personal. Soc. Psychol.* 60 456–470. 10.1037/0022-3514.60.3.456 2027080

[B21] Hewitt P. L. Flett G. L. Ediger E. (1996). Perfectionism and depression: longitudinal assessment of a specific vulnerability hypothesis. *J. Abnorm. Psychol.* 105 276–280. 10.1037/0021-843X.105.2.276 8723009

[B22] Holmbeck G. N. (1997). Toward terminological, conceptual, and statistical clarity in the study of mediators and moderators: examples from the child-clinical and pediatric psychology literatures. *J. Consult. Clin. Psychol.* 65 599–610. 10.1037//0022-006x.65.4.599 9256561

[B23] Hu L. Bentler P. M. (1999). Cutoff criteria for fit indexes in covariance structure analysis: conventional criteria versus new alternatives. Structural equation modeling. *Multidiscip. J.* 6 1–55. 10.1080/10705519909540118

[B24] Karaś D. Cieciuch J. Negru O. Crocetti E. (2015). Relationships between identity and well-being in italian, polish, and romanian emerging adults. *Soc. Indic. Res.* 121 727–743. 10.1007/s11205-014-0668-9

[B25] Karaś D. Kłym M. Cieciuch J. (2013). Eudajmonistyczny dobrostan psychiczny a kształtowanie poczucia tożsamości w sferze edukacyjnej i zawodowej [Eudaimonic well-being and identity formation in the educational and vocational domains; in Polish]. *Psychol. Rozwojowa* 18 87–101. 10.4467/20843879PR.13.006.1018

[B26] Klimstra T. A. Denissen J. J. A. (2017). A theoretical framework for the associations between identity and psychopathology. *Dev. Psychol.* 53 2052–2065. 10.1037/dev0000356 29094969

[B27] Klimstra T. A. Luyckx K. Goossens L. Teppers E. De Fruyt F. (2013). Associations of identity dimensions with big five personality domains and facets. *Eur. J. Personal.* 27 213–221. 10.1002/per.1853

[B28] Luyckx K. Schwartz S. J. Goossens L. Beyers W. Missotten L. (2011). “Processes of personal identity formation and evaluation,” in *Handbook of Identity Theory and Research*, eds Schwartz S. J. Luyckx K. Vignoles V. L. (New York: Springer), 77–98. 10.1007/978-1-4419-7988-9-4

[B29] Luyckx K. Schwartz S. J. Goossens L. Pollock S. (2008a). Employment, sense of coherence, and identity formation: contextual and psychological processes on the pathway to sense of adulthood. *J. Adolesc. Res.* 23 566–591. 10.1177/0743558408322146

[B30] Luyckx K. Schwartz S. J. Goossens L. Soenens B. Beyers W. (2008b). Developmental typologies of identity formation and adjustment in female emerging adults: a latent class growth analysis approach. *J. Res. Adolesc.* 18 595–619. 10.1111/j.1532-7795.2008.00573.x

[B31] Luyckx K. Schwartz S. J. Berzonsky M. D. Soenens B. Vansteenkiste M. Smits I. (2008c). Capturing ruminative exploration: extending the four-dimensional model of identity formation in late adolescence. *J. Res. Personal.* 42 58–82. 10.1016/j.jrp.2007.04.004

[B32] Luyckx K. Soenens B. Goossens L. Beckx K. Wouters S. (2008d). Identity exploration and commitment in late adolescence: correlates of perfectionism and mediating mechanisms on the pathway to well–being. *J. Soc. Clin. Psychol.* 27 336–361. 10.1521/jscp.2008.27.4.336

[B33] Marcia J. E. (1966). Development and validation of ego-identity status. *J. Personal. Soc. Psychol.* 3 551–558. 10.1037/h0023281 5939604

[B34] Muthén L. K. Muthén B. O. (1998-2012). *Mplus User’s Guide*, 7th Edn. Los Angeles, CA: Muthén & Muthén

[B35] Piotrowski K. (2013). Identity in adolescence and emerging adulthood: relationships with emotional and educational factors. *Pol. Psychol. Bull.* 3 266–276. 10.2478/ppb-2013-0030

[B36] Piotrowski K. (2018). Adaptation of the utrecht-management of identity commitments scale (U-MICS) to the measurement of the parental identity domain. *Scand. J. Psychol.* 59 157–166. 10.1111/sjop.12416 29231970

[B37] Piotrowski K. (2019). *Dimensions of Parental Identity and Their Relationships with Adult Attachment and Perfectionism in Mothers.* Poland: University of Social Sciences and Humanities 10.31234/osf.io/ka6pz

[B38] Piotrowski K. Bojanowska A. (2019). Factor structure and psychometric properties of a polish adaptation of the frost multidimensional perfectionism scale. *Curr. Psychol.* 1–10. 10.1007/s12144-019-00198-w

[B39] Piotrowski K. Brzezińska A. I. (2017). Skala wymiarów rozwoju tożsamości DIDS: wersja zrewidowana [the dimensions of identity development scale (DIDS): the revised polish adaptation; in polish]. *Psychol. Rozwojowa* 22 89–111. 10.4467/20843879PR.17.024.8070

[B40] Piotrowski K. Brzeziñska A. I. (2018). A cluster-analytic approach to identity style classification: four seems to be better than three. *Eur. J. Dev. Psychol.* 16 522–537. 10.1080/17405629.2018.1467755

[B41] Satorra A. Bentler P. M. (1994). “Corrections to test statistics and standard errors in covariance structure analysis,” in *Latent Variables Analysis: Applications for Developmental Research*, eds von Eye A. Clogg C. C. (Thousand Oaks: Sage Publications), 399–419.

[B42] Słowińska A. Zbieg A. Oleszkowicz A. (2014). Kwestionariusz ruminacji-refleksji (RRQ) paula D. trapnella i jennifer D. campbell – polska adaptacja metody [paul D. trapnell and jennifer D. campbell’s the rumination-reflection questionnaire – polish adaptation; in polish]. *Polskie Forum Psychol.* 19 457–478.

[B43] Stöber J. Joormann J. (2001). Worry, procrastination, and perfectionism: differentiating amount of worry, pathological worry, anxiety, and depression. *Cognit. Ther. Res.* 25 49–60. 10.1023/A:1026474715384

[B44] Stoeber J. Damian L. E. (2016). “Perfectionism in employees: work engagement, workaholism, and burnout,” in *Perfectionism, Health, and Well-Being*, eds Sirois F. M. Molnar D. S. (New York: Springer), 265–284. 10.1007/978-3-319-18582-8_12

[B45] Stoeber J. Eismann U. (2007). Perfectionism in young musicians: relations with motivation, effort, achievement, and distress. *Personal. Individ. Differ.* 43 2182–2192. 10.1016/j.paid.2007.06.036

[B46] Stoeber J. Harris R. A. Moon P. S. (2007). Perfectionism and the experience of pride, shame, and guilt: comparing healthy perfectionists, unhealthy perfectionists, and non-perfectionists. *Personal. Individ. Differ.* 43 131–141. 10.1016/j.paid.2006.11.012

[B47] Stoeber J. Otto K. (2006). Positive conceptions of perfectionism: approaches, evidence, challenges. *Personal. Soc. Psychol. Rev.* 10 295–319. 10.1207/s15327957pspr1004-2 17201590

[B48] Tangney J. P. Wagner P. E. Hill-Barlow D. Marschall D. E. Gramzow R. (1996). Relation of shame and guilt to constructive versus destructive responses to anger across the lifespan. *J. Personal. Soc. Psychol.* 70 797–809. 10.1037/0022-3514.70.4.797 8636899

[B49] Trapnell P. D. Campbell J. D. (1999). Private self-consciousness and the five-factor model of personality: distinguishing rumination from reflection. *J. Personal. Soc. Psychol.* 76 284–304. 10.1037/0022-3514.76.2.284 10074710

[B50] van Doeselaar L. Klimstra T. A. Denissen J. J. A. Branje S. Meeus W. (2018). The role of identity commitments in depressive symptoms and stressful life events in adolescence and young adulthood. *Dev. Psychol.* 54 950–962. 10.1037/dev0000479 29251964

[B51] Vondracek F. W. Schulenberg J. Skorikov V. Gillespie L. K. Wahlheim C. (1995). The relationship of identity status to career indecision during adolescence. *J. Adolesc.* 18 17–29. 10.1006/jado.1995.1003 9819934

[B52] Vosylis R. Erentaitë R. Crocetti E. (2018). Global versus domain-specific identity processes: which domains are more relevant for emerging adults? *Emerg. Adulthood* 6 32–41. 10.1177/2167696817694698

